# Psychometric Properties of the Diagnostic Interview for Sexual Dysfunctions in Women in a Symptom-Reporting Sample

**DOI:** 10.1177/10731911241253659

**Published:** 2024-06-03

**Authors:** Rebekka Schwesig, Maike Borchardt, Julia Velten, Jürgen Hoyer

**Affiliations:** 1 Technische Universität Dresden, Germany; 2 Ruhr University Bochum, Germany

**Keywords:** DISEX-F, DSM-5, ICD-11, structured interview, sexual dysfunctions, psychometric properties

## Abstract

While structured clinical interviews are considered the gold standard for diagnosing mental disorders, respective instruments are still lacking in the field of sexual dysfunctions. The study evaluates the psychometric properties of the new *Diagnostic Interview for Sexual Dysfunctions in Women* (DISEX-F), which is based on the eleventh edition of the *International Statistical Classification of Diseases* (ICD-11) and the fifth edition of the *Diagnostic and Statistical Manual of Mental Disorders* (DSM-5), in a sample of 100 women with self-reported sexual problems. Participants were interviewed twice by trained diagnosticians with the DISEX-F. A third diagnostician evaluated the audio records of the initial interview. Participants also completed self-report measures of sexual functioning/distress and interview acceptance. The DISEX-F demonstrates excellent inter-rater reliability, good test-retest reliability, and strong convergent and discriminant evidence of validity. Furthermore, it achieves high acceptance among participants. Discordant diagnostic outcomes were especially linked to false differential diagnostic decisions and information variance in participants reporting. The results strongly support using the DISEX-F for women presenting with self-reported sexual problems in practice and research.

The development of structured interviews, such as the Composite International Diagnostic Interview (CIDI; World Health Organization [WHO], 1997) and the Structured Clinical Interview for DSM-V (SCID-5; First et al., 2016), has played a crucial part in enhancing reliability and validity of psychiatric diagnoses and has enabled the growth of epidemiologic research in psychiatry in the past decades (Grove et al., 1981; Kessler, 2007). Today, the use of structured interviews is considered a necessary component of evidence-based practice and regarded as the gold standard for the diagnosis of mental disorders in practice and research (see Jensen-Doss, 2015; Rettew et al., 2009).

However, as none of the existing structured instruments include criteria for sexual dysfunctions, such a gold-standard instrument is still missing for the field of sexual dysfunctions. This has significant problematic effects: Due to a high variability in applied diagnostic methods, research results on sexual dysfunctions are difficult to compare across studies (McCabe et al., 2016). Moreover, sexual dysfunctions are often not systematically assessed in routine care which leads to low numbers of assigned diagnoses and treatments, despite prevalence rates actually being high (Ribeiro et al., 2014; Velten et al., 2021b; Zannoni et al., 2021): According to recent representative population-based studies, 18%–46% of women experience problems related to sexual desire, arousal, orgasm, or pain in the past 12 months. Among these, 4%–18% of women meet the diagnostic criteria for sexual dysfunctions (Briken et al., 2020; Mitchell et al., 2016).

In response to this gap, the Diagnostic Interview for Sexual Dysfunctions in Women (DISEX-F) was developed (Schwesig, Velten, & Hoyer, 2022). This instrument allows a structured diagnostic assessment of female sexual dysfunctions aligning with diagnostical criteria of both current classification systems, that is, the fifth edition of the Diagnostic and Statistical Manual of Mental Disorders (DSM-5, American Psychiatric Association [APA], 2013) and the eleventh edition of the International Statistical Classification of Diseases (ICD-11, WHO, 2019). The DISEX-F is a freely accessible and economical tool developed for use by experienced clinicians as well as non-specialized clinical researchers and practitioners with little or no clinical experience in diagnosing and treating sexual dysfunctions. In their initial validation study, Schwesig et al. (2023a) tested the DISEX-F using a vignette-based validation method involving standardized patients (actresses portraying predefined diagnostic case vignettes). In this study, the sensitivity of the DISEX-F was generally found to be clinically satisfactory (DSM-5: .90–.99; ICD-11: .95–.99). However, both sensitivity (DSM-5: .40–.92; ICD-11: .71–.96) and inter-rater reliability (DSM-5: Cohen’s kappa [κ] = .44–1; ICD-11: κ = .75–.94) showed significant variation across disorders and classification systems. A subsequent error analysis identified imprecise acting and incorrect differential diagnostic decisions as the primary causes of mismatches. The DISEX-F received widespread acceptance among the diagnosticians, affirming its practical utility. These initial research results on the ICD-11 disorders in the DISEX-F strongly advocate for the adoption of the DISEX-F in practice and research, supported by its high acceptance rate and excellent psychometric properties for ICD-11 diagnoses. However, the findings regarding DSM-5 diagnoses were mixed, which the authors trace back to a lack of clarity in the DSM-5 criteria as well as higher demands on clinical knowledge and experience (see for further explanation, Schwesig et al., 2023a). The vignette-based approach offers distinct advantages for instrument development such as easy inclusion of both prototypical and rare patient profiles, as well as precise control over the ratio of cases to non-cases and the individual prevalence of specific disorders. However, the authors conclude that this may also have led to a biased estimation of reliability and validity and, therefore, to limited ecological validity. While this study marked the initial step of the validation of the DISEX-F under ideal conditions, it is crucial to continue the validation process by assessing its clinical validity by involving women with sexual problems.

To be applicable in clinical practice, the DISEX-F must—in addition to demonstrating reliability and validity—fulfill the criteria of feasibility and patient and interviewer acceptance (Pinninti et al., 2003). While interviewer acceptance has been explored in the initial validation study, the acceptance of the DISEX-F among women experiencing sexual problems has not been investigated so far. Clinicians commonly express skepticism regarding the acceptance of structured interviews among patients. Concerns raised include apprehensions about potential interference with the therapeutic relationship and the significant time commitment demanded from both patients and clinicians, leading to doubts about its superiority over clinical judgment (Aboraya, 2009; Bjaastad et al., 2019; Marshall et al., 2001). Despite the considerable benefits associated with structured interviews, most clinicians rely on unstandardized clinical interviews when diagnosing patients (Jensen & Weisz, 2002; Miller et al., 2001; Rettew et al., 2009). To address these concerns, an important aim of this study is to explore how participants perceive the DISEX-F interview process.

## The Present Study

To take the next essential steps in the validation process of the DISEX-F, we aim to further investigate its validity and reliability in sample of women with self-reported sexual problems. Furthermore, we aim to assess the acceptance of the DISEX-F among participants. Next to inter-rater reliability, we assess test-rest reliability (the “most stringent test of diagnostic reliability,” Grove et al., 1981) as well as evaluate convergent and discriminant evidence of validity. Furthermore, to gain an understanding of what causes diagnostical disagreement when using the DISEX-F, the source of discordant diagnostic outcomes will be analyzed. We will use several screening measures for mental disorders to comprehensively describe the clinical attributes of our validation sample and, thus, to enhance replicability.

To assess convergent and discriminant evidence of validity, we choose three frequently used construct-related self-report measures for comparison with the DISEX-F results: That is, (a) the Female Sexual Functioning Index (FSFI, Rosen et al., 2000) assessing overall level of sexual functioning, (b) the Sexual Interest and Desire Inventory-Female-Self-Report (SIDI-F-SR, Velten et al., 2021a) measuring severity of Hypoactive Sexual Desire Dysfunction in women, and (c) the Female Sexual Distress Scale-Desire/Arousal/Orgasm (FSDS-DAO, Derogatis et al., 2021) measuring sexual distress in women. Although none of these scales provides categorical diagnostic decision rules for sexual dysfunctions according to DSM or ICD, research provided convergent and discriminant evidence of their validity: The FSFI total score and each of the domain scores discriminate significantly between groups of women with and without sexual dysfunction (Meston, 2003; Rosen et al., 2000; Wiegel et al., 2005). The revised version of FSDS (FSDS-R; a previous, two-item shorter version of the FSDS-DAO) successfully distinguishes between sexually functional and dysfunctional women (Derogatis et al., 2008) and between women with Hypoactive Sexual Desire Dysfunction and without (Derogatis et al., 2011). The SIDI-F discriminates between women with Hypoactive Sexual Desire Dysfunction and women without sexual dysfunctions (Clayton et al., 2006, 2010). In line with the research results mentioned earlier, we expect the presence of a DSM-5 and ICD-11 diagnosis in the DISEX-F to be negatively associated with the FSFI total score and positively associated with the FSDS-DAO total score. In comparison with the ICD-11, the DSM-5 has more exclusion criteria, which the self-report questionnaires do not account for (e.g., symptom duration of at least 6 months, symptoms not caused by organic etiology, medication, drugs, relationship distress or significant stress; see for an overview: Schwesig, Briken, et al., 2022). Thus, we expect both associations to be significantly higher for ICD-11 diagnoses than for DSM-5 diagnoses. In accordance with research results from Clayton et al. (2006, 2010), we expect a significant negative association between the presence of Hypoactive Sexual Desire Dysfunction (ICD-11) in the DISEX-F and the SIDI-F-SR total score. To further estimate convergent and discriminant evidence of validity on a disorder level, we explore the association of the desire, arousal, orgasm, lubrication, and pain subscale of the FSFI with every ICD-11 and DSM-5 diagnosis in the DISEX-F. In addition, we explore the association between the SIDI-F-SR total score with all other ICD-11 and DSM-5 diagnoses in the DISEX-F. In accordance with Schwesig et al. (2023a) and following the Consensus based Standards for the Selection of Health Measurment Instruments ([COSMIN] guidelines, see Mokkink et al., 2010), we established predetermined minimum thresholds for the psychometric criteria of the DISEX-F. These thresholds serve as the minimum requirements for the instrument to be seen as clinically reliable and valid for diagnostic purposes (see Methods section for a detailed description of the set statistical margins).

## Methods

The study received ethical approval from the ethics committee of the Technische Universität Dresden (SR-EK-42012023) and was preregistered before data collection (see osf.io/q8d36).

### Participants

We aimed for a sample size of at least *N* = 94 women experiencing sexual problems (see pre-registration for a detailed description on how sample size was determined). To recruit a sample of women representing the heterogeneity of different ages, sexual orientations, and ethnicities across the general population, a range of recruitment strategies were used, for example, posts on social media, announcements on local radio stations and newspapers, as well as through leaflets distributed at specialized counseling agencies. Participants were required to be at least 18 years of age, be fluent in German, to identify as female or non-binary, and to have female sexual organs either since birth or through gender-reassignment surgery. Furthermore, participants had to be currently experiencing self-identified sexual problems related to sexual desire, arousal, orgasm, or sexual pain. For participating in the study, individual feedback on the results of the diagnostical assessment as well as information on help and treatment services were offered. There were no additional exclusion criteria for participating. All participants included in the study (*N* = 100, *M_age_* = 36.36, *SD* = 12.21, range 19–72) identified as cis-women and reported to have female sexual organs since birth (see Table S1 in the Supplemental Materials for further sample characteristics).

### Diagnosticians

Diagnosticians were all women and clinical psychologists (*n* = 1) or advanced master students of clinical psychology (*n* = 3). Three of four diagnosticians had also participated in the initial validation study of the DISEX-F (Schwesig et al., 2023a) as diagnosticians. All diagnosticians were required to complete a systematic and standardized training beforehand (see Schwesig et al., 2023a). Diagnosticians conducted between 32 and 81 interviews (*M* = 50, *SD* = 20.92) during this study.

### Material and Measurements

#### DISEX-F for DSM-5 and ICD-11

The DISEX-F (Schwesig, Velten, & Hoyer, 2022) is a semi-standardized clinical interview designed for the precise evaluation and categorical diagnosis of sexual dysfunctions, suitable for both clinical practice and research settings. The interview is closely aligned with the content and terminology of the DSM-5 and ICD-11 and enables evaluation according to either the DSM-5 or ICD-11 criteria. To this end, the DISEX-F includes two distinct evaluation sheets. Moreover, it enables the identification of specific subtypes of sexual dysfunctions (generalized vs situational, lifelong vs acquired). In its female version (DISEX-F), the tool encompasses all diagnostic criteria for the following ICD-11 disorder categories: Hypoactive Sexual Desire Dysfunction, Female Sexual Arousal Dysfunction, Orgasmic Dysfunction, and Sexual Pain-Penetration Disorder. In addition, following DSM-5 criteria, it covers the Female Sexual Interest/Arousal Disorder, the Female Orgasmic Disorder, and the Genito-Pelvic Pain/Penetration Disorder. The DISEX-F is freely accessible in its original German version as well as in a preliminary English translation at osf.io/E86HP.

#### Female Sexual Functioning Index

The FSFI (Rosen et al., 2000) is a self-report measure for female sexual functioning (assessed time period: past 4 weeks). The 19-item measure consists of six separate domains, namely desire, arousal, lubrication, orgasm, satisfaction, and pain. Items are rated on a 5-point Likert-type scale with a total score ranging from 0 to 36. Higher scores indicate higher sexual functionality. To include women without a sexual partner, we added a zero option to Item 15 to indicate “no sexual partner.” The initial validation of the FSFI indicated excellent internal reliability (Cronbach’s alpha [α] = .97 for the total scale, α≥ .89 for all domains) and good test-retest reliability (*r* = .79–.86; Rosen et al., 2000). In the present sample, the internal consistency of the FSFI was α = .93 for the total scale, α = .88 for the desire domain, α = .95 for the arousal domain, α = .95 for the lubrication domain, α = .94 for the orgasm domain, α = .59 for the satisfaction domain, and α = .96 for the pain domain. For an extensive review of numerous additional psychometric studies on the FSFI, see the work of Neijenhuijs et al. (2019).

#### Female Sexual Distress Scale-Desire/Arousal/Orgasm

The FSDS-DAO (Derogatis et al., 2021) is a self-report measure of sexually related personal distress in women (assessed time period: past 30 days). It retains the 13 items from FSDS-R (Derogatis et al., 2002, 2008) but also includes two additional items assessing distress related to arousal and orgasm. Items are rated on a 5-point Likert-type scale with a total score ranging from 0 to 60. Higher scores indicate greater distress. Like the FSDS-R, the FSDS-DAO has been shown to have a high degree of internal consistency (α = .91–.97) and test-retest reliability (Derogatis et al., 2008, 2011, 2021). In the present sample, the internal consistency of the FSDS-DAO was α = .92.

#### Sexual Interest and Desire Inventory-Female

The SIDI-F-SR (Velten et al., 2021b) is a 13-item self-report version of the SIDI-F (Clayton et al., 2006), a measure of hypoactive sexual desire dysfunction severity in women (covered time period: past 4 weeks). Item scores can be summed for a total score ranging from 0 to 51, with higher scores indicating higher levels of sexual desire. The SIDI-F shows excellent internal consistency (Cronbach’s alpha of .90). The internal consistency (α = .76) as well as the convergent evidence of validity of the SIDI-F-SR are comparable to the SIDI-F, and results of both measures highly agree with each other (Velten et al., 2021a). The internal consistency of the SIDI-F-SR in the present sample was α = .82.

#### Patient Acceptance Questionnaire

To assess the participants’ evaluation of the interview itself, we used the Patient Acceptance Questionnaire (PAQ; Suppiger et al., 2009). The PAQ assesses the patient’s acceptance of the structured interview (10 items, 4-point Likert-type scale ranging from 0 = *disagree* to 3 = *completely agree*) as well as their global satisfaction with the structured interview on a rating scale from 0 = *not at all satisfied* to 100 = *totally satisfied.* The internal consistency of the questionnaire was α = .43 in the present study sample compared to α = .50 in the study sample by Suppiger et al. (2009).

#### Screening for Mental Disorders

##### Patient Health Questionnaire

The Patient Health Questionnaire (PHQ; Spitzer et al., 1999) is a self-report instrument for the assessment of symptoms associated with mental health disorders. For sample description in this study, we especially use data and cutoffs of the following PHQ modules: somatoform disorders (PHQ-15, Kroenke et al., 2002; cutoff ≥10: Körber et al., 2011), depressive disorders (PHQ-9; Kroenke et al., 2001; cutoff ≥10: Negeri et al., 2021), and anxiety disorders (Generalized Anxiety Disorder Scale 7 (GAD-7); Spitzer et al., 2006; cutoff ≥8: Plummer et al., 2016). In addition, responses to questions related to panic disorders, eating disorders, and alcohol abuse were also evaluated. Psychometric properties of the PHQ and its modules are well established and have been assessed in various original studies and meta-analyses (e.g., Gilbody et al., 2007; Kroenke et al., 2010; Plummer et al., 2016; Spitzer et al., 1999, 2000). In the present study sample, the internal consistency was α = .76 for the PHQ-15, α = .77 for the PHQ-9, and α = .77 for the GAD-7.

##### Short Screening Scale for DSM-4 Posttraumatic Stress Disorder

The Short Screening Scale for DSM-4 Posttraumatic Stress Disorder (SSS; Breslau et al., 1999) is a short screening scale for DSM-4 (WHO, 1994) posttraumatic stress disorder (PTSD). The seven items are answered on a 4-point Likert-type scale (1 = *not at all*, 5 = *five times per week/almost always*). A score of 4 or more on the scale shows a sensitivity of 80% and a specificity of 97% in diagnosing DSM-4 PTSD (Breslau et al., 1999). In this study, participants completed the SSS if they had affirmatively responded to the PTSD screening question of the Composite International Diagnostic-Screener (CID-S, Wittchen et al., 1999). This question assesses exposure to an actual or threatened event or situation, as required by the *A-Criterion* for PTSD in the DSM-5. The internal consistency of the SSS was α = .79 in the present study sample.

### Procedure

Prior to their participation in the study, participants provided their informed consent. An online questionnaire, filled in before the study appointments, assessed the fulfillment of the inclusion criteria. The presence of self-reported sexual problems was queried using the four screening questions of the DISEX-F asking about recent problems with sexual desire, arousal, orgasm, and sexual pain. A positive response to at least one of the four screening questions was required for participation. Upon meeting all inclusion criteria, participants proceeded with the online questionnaire which included various demographic questions, the PHQ, an adapted version of the SSS, and the self-report version of the DISEX-F (DISEX-F-SR, Schwesig et al., 2023b; please note that the results from the DISEX-F-SR are not presented here). After completion, participants were contacted to arrange two study appointments.

During the first in-person study appointment, participants underwent two procedures: (a) a face-to-face interview conducted by a trained diagnostician using the DISEX-F, followed by the completion of the PAQ, and (2) filling out the FSFI, SIDI-F, and FSDS-DAO questionnaires (with the order of the scales randomized for each patient; the sequence in which participants were interviewed with the DISEX-F and completed the three sexual functioning questionnaires was balanced between participants). After the appointment, diagnosticians evaluated the interview according to both ICD-11 and DSM-5 criteria. The first DISEX-F interview was audio-recorded, and these recordings were evaluated by a trained diagnostician who was blinded to the results of the first interview evaluation.

The second study appointment took place on average 11 days after the first study appointment (*Median* = 9, *M* = 10.67, *SD* = 5.14, range = 2–40 days) and included the re-administering of the DISEX-F by a third diagnostician who was blinded to the results of the first interview and to the evaluation of the recorded audio tape. Initial interviews lasted on average 38 min (*SD* = 13 min, range: 9–69 min), second interviews lasted on average 34 min (*SD* = 10 min, range: 9–56 min).

### Statistical Analyses

We predefined lower limits for the magnitude of psychometric criteria of the DISEX-F that the diagnostic instrument should at least reach to be considered clinically reliable and valid: Like in the initial validation study of the DISEX-F (Schwesig et al., 2023a), we defined κ=.40 as the minimum acceptable threshold for inter-rater reliability and test-retest reliability. In addition to considering practical feasibility constraints related to the maximum sample size, this minimum threshold was chosen primarily because it conforms to the established convention of categorizing kappa values of κ≥.40 as indicative of moderate agreement (see Landis & Koch, 1977). As a measure for convergent evidence of validity, we calculate receiver operating characteristic (ROC) curves and the corresponding area under the curve (AUC) values. We specified a minimum acceptable threshold for the AUC values of >.5. Accordingly, there had to be more than 50% probability that a randomly selected clinical case in the DISEX-F scored higher (or lower) on a specific self-report questionnaire than a randomly selected non-case. To meet the pre-established minimum requirements, it was imperative that the lower limit of the confidence interval (CI) of the calculated estimate did not fall below the defined minimum threshold.

In line with Schwesig et al. (2023a), inter-rater reliability and test-retest reliability were computed individually for each sexual dysfunction in the DSM-5 and ICD-11 using Cohen’s kappa estimates. We calculated one-sided 95% CIs to identify precision of the calculated estimates and to test our hypotheses. To this end, the two-sided confidence level was technically set to .90, but only the lower limit was evaluated while setting the upper limit to 1. Due to the malfunction of the audio recording during one interview, the calculation of the inter-rater reliability was based on *n* = 99 participants. Like Schwesig et al. (2023a), we examined the sources of discordant diagnostic outcomes found between assigned diagnoses of the first interview and the audio record as well as between assigned diagnoses of the first and the second interviews in a subsequent analysis. For this purpose, we used the checklist with error categories from Schwesig et al. (2023a) and extended it to code mismatch that occurred due to discrepancies in patient’s answers. This checklist was based on error categories originally identified by other validation studies of structured interviews (Neuschwander et al., 2013; Suppiger et al., 2008). We identified the main source of mismatch for each discordant diagnostic outcome.

Convergent and discriminant evidence of validity of the DISEX-F was assessed by comparing the DISEX-F results of the first interview (ICD-11 and DSM-5 diagnoses) to the total scores of the FSFI, SIDI-F-SR, and FSDS-DAO. To assess the magnitude of the associations as well as their significance, we calculated ROC curves, the corresponding AUC values, as well as their 95% CIs. To test the hypotheses on the magnitude of two anticipated associations, we compared the AUC values of the corresponding ROC curves using the “bootstrap” and “delong” method from the “pROC” package (version 1.18.0, Robin et al., 2011). As suggested by Meston et al. (2020), in order to avoid bias in the FSFI scores toward low sexual functioning and, thus, bias in our results, we recalculated all analyses involving FSFI scores within a subsample of exclusively sexually active women who had not indicated a zero score (“no sexual activity”) on any of the FSFI items.

We calculated the mean and standard deviations as well as the distribution of given answers for each item of the PAQ to assess the acceptance of the DISEX-F among participants. Data of the study as well as the analysis code to reproduce all statistical results in R are freely accessible at osf.io/wj75g.

## Results

### Clinical Characteristics of the Sample

Twenty-six individuals met the cutoffs of the PHQ for depressive disorders (*M* = 6.65, *SD* = 4.22; range = 0–19), 21 for anxiety disorders (*M* = 4.73, *SD* = 2.97; range = 0–12), 36 for somatic symptom disorders (*M* = 7.79, *SD* = 4.38; range = 1–17), and 14 for PTSD (*M* = 0.99, *SD* = 1.77; range = 0–6). Furthermore, based on the PHQ evaluation algorithm, one participant showed signs of binge eating disorder, and seven participants exhibited symptoms of alcohol abuse.

### Inter-Rater Reliability

Inter-rater reliability was calculated to assess the agreement of diagnoses assigned directly after the first DISEX-F interview and the diagnoses assigned based on the audio record of that interview. Kappa estimates were excellent for all diagnoses in both classification systems (κ≥.87; “almost perfect” according to Landis & Koch, 1977), and their lower CIs were all ≥.79 and, therefore, within a clinical satisfactory range (see Table 1).

**Table 1:** Inter-Rater Reliability (n = 99) and Test-Retest Reliability (N = 100) of the DSM-5 and ICD-11 Diagnoses in the DISEX-F

	**Inter-rater reliability**				**Test-retest reliability**			
	Frequencies		%	Cohen’s kappa	Frequencies		%	Cohen’s kappa
**Diagnoses**	−/− +/−	−/+ +/+			−/− +/−	−/+ +/+		
**DSM-5**								
Female Sexual Interest/Arousal Disorder	57 1	4 37	95	.89 [.82, .97]	52 6	9 33	85	.69 [.57, .81]
Female Orgasmic Disorder	57 1	2 39	97	.94 [.88, 1.00]	49 3	11 37	86	.72 [.60, .83]
Genito-Pelvic Pain/Penetration Disorder	58 3	2 36	95	.89 [.82, .97]	56 7	5 32	88	.75 [.63, .86]
**ICD-11**								
Hypoactive Sexual Desire Dysfunction	70 0	0 29	100	1.00 [1.00, 1.00]	56 1	15 28	84	.66 [.54, .78]
Female Sexual Arousal Dysfunction	73 0	0 26	100	1.00 [1.00, 1.00]	65 3	8 24	89	.74 [.61, .86]
Orgasmic Dysfunctions	56 1	0 42	99	.98 [.95, 1.00]	46 3	11 40	86	.72 [.61, .83]
Sexual Pain-Penetration Disorder	59 3	3 34	94	.87 [.79, .95]	58 5	5 32	90	.79 [.68, .89]

*Note.*−/− = correct negative, −/+ false negative, +/− false positive, +/+ = correct positive; % = percentage of agreement; Brackets indicate one-sided 95% confidence intervals.

### Test-Retest Reliability

Test-retest reliability was calculated to assess the agreement of diagnoses between the first and the second interview. All found kappa estimates indicated at least substantial agreement (κ≥.66; Landis & Koch, 1977), and their corresponding CIs fell within a clinical acceptable range (all lower CI limits were ≥.54). Remarkably, the Genito-Pelvic Pain/Penetration Disorder (DSM-5) and the Sexual Pain-Penetration Disorder (ICD-11) exhibited the highest kappa estimates. In contrast, the Female Sexual Interest/Arousal Disorder (DSM-5) and the Hypoactive Sexual Desire Dysfunction (ICD-11) demonstrated the lowest kappa estimates (see Table 1). Overall, diagnosticians assigned more diagnoses during the second interview than during the first one (see Table S2 in the Supplemental Materials).

### Source of Mismatch

#### Between Diagnoses Based on the First Interview and Diagnoses Based on the Audio Record

Mismatch between diagnostic evaluation of the first interview and the audio record was rare: 2.2% of DSM-5 evaluations, that is, 13 out of 600 (100 cases * 2 raters * 4 disorders), and 0.9% of ICD-11 evaluations, that is, 7 out of 800 (100 cases * 2 raters * 3 disorders) diagnostic decisions deviated from one another. Subsequent analyses of the few discordant diagnostic outcomes indicated that the mismatch between diagnoses could be mainly traced back to divergent differential diagnostic decisions made by the diagnosticians. This source of mismatch accounted for 53.8% (DSM-5) and 57.1% (ICD-11) of discordant diagnostic outcomes. Other identified sources of mismatch were logging errors or incorrect application of evaluation rules regarding symptom severity, duration, or distress (ICD-11: 28.6%; DSM-5: 38.5%; see Table 2 for a full report). Incorrect differential diagnostic decisions, for example, assigning the Sexual Pain-Penetration Disorder despite the medical diagnosis of vestibulodynia, were rare.

**Table 2:** Source of Mismatch Between the Diagnoses of the First DISEX-F Interview and the Evaluated Audio Record.

**Type of error**	**DSM-5**					**ICD-11**					
	IA	O	P	In total	%	I	A	O	P	In total	%
**Incorrect or divergent evaluation**											
Incorrect logging or incorrect application of K or M-criteria^b^	3	1	1	5	38.5	0	0	1	1	2	28.6
Incorrect differential diagnostic decision of one diagnostician	1	0	0	1	7.7	0	0	0	1	1	14.3
Divergent differential diagnostic decisions	1	2	4	7	53.8	0	0	0	4	4	57.1
**In total**	**5**	**3**	**5**	**13^a^**	**100**	**0**	**0**	**1**	**6**	**7^a^**	**100**

*Note.* IA = *Female Sexual Interest/Arousal Disorder*; I = *Hypoactive Sexual Desire Dysfunction*; A = *Female Sexual Arousal Dysfunction*; O = *Female Orgasmic Disorder* (DSM-5) or *Orgasmic Dysfunctions* (ICD-11), P = *Genito-Pelvic Pain/Penetration Disorder* (DSM-5) *or Sexual Pain-Penetration Disorder* (ICD-11).

a There were *n* = 600 (DSM-5; 100 cases * 2 rater * 3 disorders) and *n* = 800 (ICD-11; 100 cases * 2 rater * 4 disorders) possibilities for the occurrence of errors. ^b^ That is, incorrect application of evaluation rules regarding symptom severity, duration, or distress, for example, assign IA although only presence of one or two of the three necessary symptoms.

#### Between Diagnoses Based on the First Interview and Diagnoses Based on the Second Interview

In total, 6.5% of DSM-5 evaluations, that is 41 out of 600 (100 cases * 2 raters * 4 disorders) and 6.4% of ICD-11 evaluations, that is 51 out of 800 (100 cases * 2 raters * 3 disorders) diagnostic decisions differed from one another. Further analyses of discordant diagnostic outcomes between the first and second interview unveiled information variance as a predominant factor: 73.2% of the mismatches in DSM-5 diagnoses and 92.2% of the mismatches in ICD-11 diagnoses could be attributed to variations in the information reported by the participants. For example, participants demonstrated inconsistencies in their responses to the screening questions and provided different information concerning the duration, frequency, or severity of symptoms. There was a trend to report elevated symptom severity, frequency, and symptom burden at the second interview compared to the first. Mismatches that occurred due to factors unrelated to the participants but related to the diagnosticians, such as incorrect interview conduction or erroneous or divergent interview evaluation, were relatively insignificant compared to the discrepancies arising from participant responses. However, the necessity to scrutinize the etiology of a sexual problem, pertinent to all DSM-5 diagnoses and the Sexual Pain-Penetration Disorder of the ICD-11, accounted for 7.8% of mismatch in the case of ICD-11 and a substantial 22.0% of mismatch in case of DSM-5 diagnoses (see Table 3 for a full report).

**Table 3:** Source of Mismatch Between the Diagnoses of the First and Second DISEX-F Interview

**Type of error**	**DSM-5**					**ICD-11**					
	IA	O	P	In total	%	I	A	O	P	In total	%
**Interview error (in total)**	**0**	**1**	**0**	**1**	**2.4**	**0**	**0**	**0**	**0**	**0**	**0**
**Incorrect or divergent evaluation (in total)**	**3**	**3**	**4**	**10**	**24.4**	**0**	**0**	**0**	**4**	**4**	**7.8**
Incorrect logging or incorrect application of K or M-criteria^b^	0	0	0	0	0	0	0	0	0	0	0
Incorrect differential diagnostic decision of one diagnostician	1	0	0	1	2.4	0	0	0	0	0	0
Divergent differential diagnostic decisions	2	3	4	9	22.0	0	0	0	4	4	7.8
**Information variance in patient reporting (in total)**	**12**	**10**	**8**	**30**	**73.2**	**16**	**11**	**14**	**6**	**47**	**92.2**
Regarding symptom severity											
T1 higher than T2	2	0	1	3	7.3	0	2	0	1	3	5.9
T2 higher than T1	5	2	0	7	17.1	8	4	2	0	14	27.5
Regarding symptom burden											
T1 higher than T2	2	0	1	3	7.3	0	1	0	0	1	2.0
T2 higher than T1	1	4	1	6	14.6	7	2	6	1	16	31.4
Regarding the screening question											
T1: affirmed, T2: negated	1	1	0	2	4.9	1	1	1	0	3	5.9
T1: negated, T2: affirmed	0	2	1	3	7.3	0	1	2	1	4	7.8
Regarding the persistence of symptoms	1	0	1	2	4.9	0	0	0	0	0	0
Regarding the adequacy of stimulation	/	1	3	4	9.8	/	0	1	3	4	7.8
Regarding the occurrence despite sexual desire	/	/	/	/	/	/	0	2	0	2	3.9
**In total**	**15**	**14**	**12**	**41** ^a^	**100**	**16**	**11**	**14**	**10**	**51** ^a^	**100**

*Note.* IA = *Female Sexual Interest/Arousal Disorder*; I = *Hypoactive Sexual Desire Dysfunction*; A = *Female Sexual Arousal Dysfunction*; O = *Female Orgasmic Disorder* (DSM-5) or *Orgasmic Dysfunctions* (ICD-11); P = *Genito-Pelvic Pain/Penetration Disorder* (DSM-5) *or Sexual Pain-Penetration Disorder* (ICD-11); T1 = first interview, T2 = second interview.

a There were *n* = 600 (DSM-5; 100 cases * 2 rater * 3 disorders) and *n* = 800 (ICD-11; 100 cases * 2 rater * 4 disorders) possibilities for the occurrence of errors. ^b^ That is, incorrect application of evaluation rules regarding symptom severity, duration, or distress, for example, assign IA although only presence of one or two of the three necessary symptoms.

### Evidence of Validity Based on the Relationship With Other Measures

#### Association of DISEX-F Diagnoses and FSFI Results

The presence of an ICD-11 diagnosis in the DISEX-F was significantly negatively associated with the FSFI total score (AUC = .69, CI [.56, 1]): There was a 69% probability that a person with an ICD-11 diagnosis in the DISEX-F scored lower on the FSFI than a person without an ICD-11 diagnosis. However, there was no significant negative association between the presence of a DSM-5 diagnosis in the DISEX-F and the FSFI total score (AUC = .50, CI [.36, 1]). The negative association between the FSFI total score and the presence of a DISEX-F diagnosis was significantly higher for ICD-11 than for DSM-5 (*D* = 1.71, *p* = .045).

Further explorative analyses showed that the number of ICD-11 diagnoses per person and the FSFI total score correlated moderately (*r* = −.50, *p* < .001). The correlation between the number of DSM-5 diagnoses per person and the FSFI total score was small, although significant (*r* = −.30, *p* = .003).

**Figure 1.**
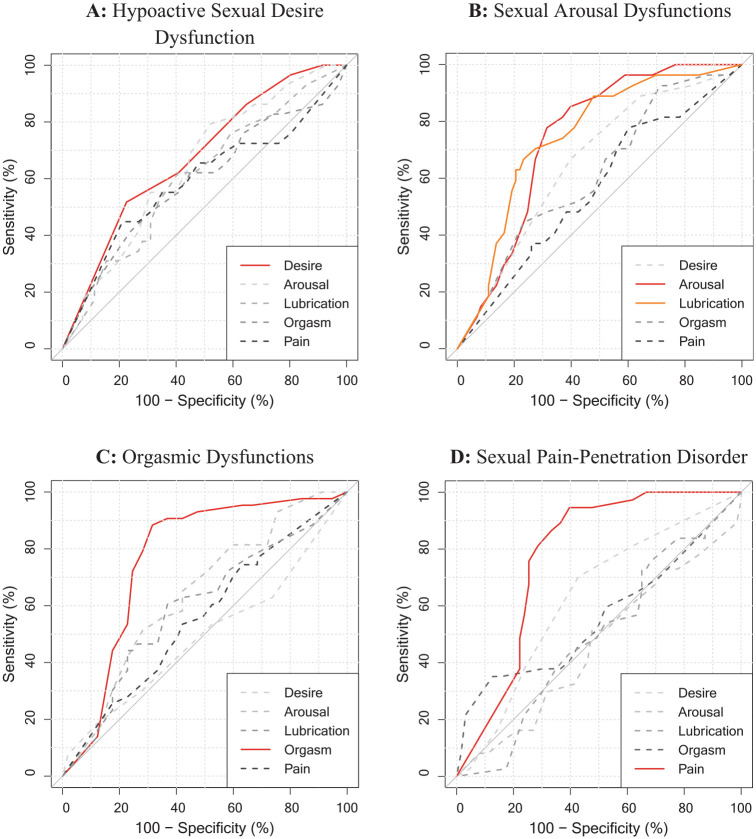
Receiver Operating Characteristic (ROC) Curves of Each FSFI-Subscales for a Specific ICD-11 Diagnosis in the DISEX-F. *Note.* Red and orange solid lines represent FSFI subscales assessing a similar construct than the present ICD-11 disorder; gray dashed lines represent subscales assessing dissimilar (somewhat more distant) constructs.

**Figure 2.**
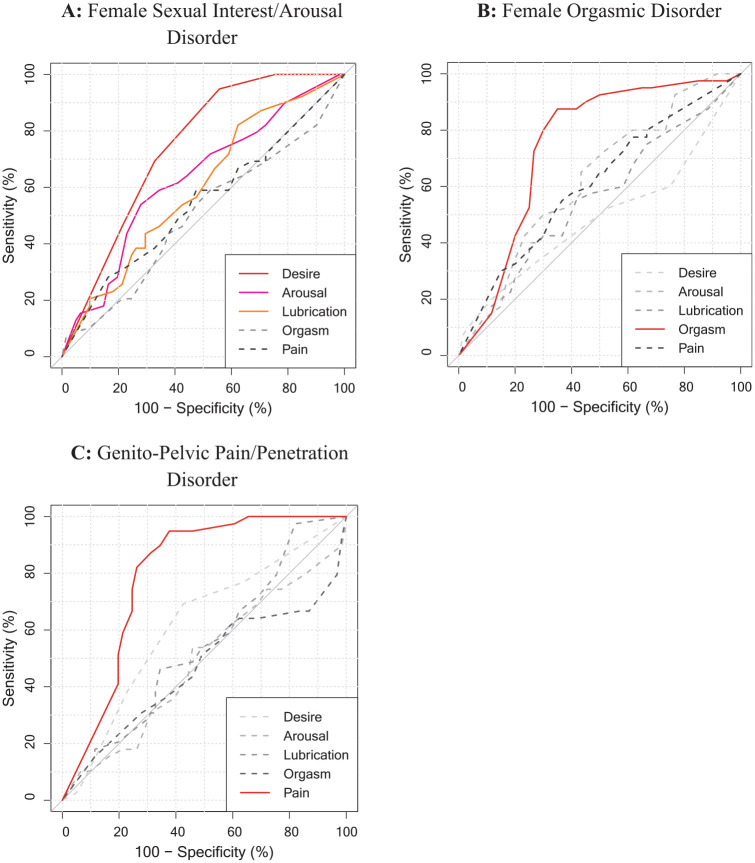
Receiver Operating Characteristic (ROC) Curves of Each FSFI-Subscales for a Specific DSM-5 Diagnosis in the DISEX-F. *Note.* Red, orange, and pink solid lines represent FSFI subscales assessing a similar construct than the present DSM-5 disorder; gray dashed lines represent subscales assessing dissimilar (somewhat more distant) constructs.

To assess convergent and discriminant evidence of validity on a disorder level, we also explored the association of every single ICD-11 and DSM-5 diagnoses in the DISEX-F with all subscales of the FSFI. Table 4 reports all computed AUC values and CIs, and Figures 1 and 2 visualizes the corresponding ROC curves. Associations yield the highest magnitudes when the diagnoses in the DISEX-F measured a construct similar to that measured by the subscales of the FSFI (e.g., DSM-5 Genito-Pelvic Pain/Penetration Disorder and FSFI subscale pain: AUC = .80). Furthermore, associations of similar constructs were descriptively higher than associations between dissimilar constructs (e.g., DSM-5 Genito-Pelvic Pain/Penetration Disorder and FSFI subscale orgasm: AUC = .47). The recalculation of the found estimates in a subsample of only sexually active women who had not indicated a zero score (“no sexual activity”) on any of the FSFI items (*n* = 70) resulted in increased associations between the ICD-11 and DSM-5 diagnoses and the FSFI total score (ICD-11: AUC = .74, CI [.61, 1]; DSM-5: AUC = .59, CI [.42, .1]). Furthermore, all estimates between DISEX-F diagnoses and construct-related FSFI subscales also increased (except for the association between FSFI arousal subscale and the Female Sexual Interest/Arousal Disorder of the DSM-5). However, a mixed picture was observed for the associations between dissimilar constructs (see Table S3 in the Supplemental Materials for a full report on all estimates).

**Table 4:** Convergent and Discriminant Evidence of Validity: AUC Values of the ROC Curves for the DISEX-F Diagnoses and the Results of the Self-Report Measures of Sexual Functioning and Distress.

	**DSM-5**			**ICD-11**			
**Self-Report Measures**	IA	O	P	I	A	O	P
**FSFI**-Desire	**.74 [.66, 1]**	.49 [.39, 1]	.62 [.52, 1]	**.68 [.58, 1]**	.66 [.56, 1]	.49 [.40, 1]	.63 [.54, 1]
**FSFI**-Arousal	**.63 [.54, .1]**	.63 [.53, 1]	.49 [.39, 1]	.65 [.55, 1]	**.74 [.66, 1]**	.64 [.55, 1]	.46 [.36, 1]
**FSFI**-Lubrication	**.60 [.50, 1]**	.55 [.46, 1]	.54 [.45, 1]	.61 [.50, 1]	**.75 [.66, 1]**	.60 [.50, 1]	.49 [.39, 1]
**FSFI**-Orgasm	.50 [.40, 1]	**.75 [.66, 1]**	.47 [.36, 1]	.59 [.48, 1]	.62 [.52, 1]	**.77 [.68, 1]**	.56 [.45, 1]
**FSFI**-Pain	.54 [.44, .1]	.61 [.51, 1]	**.80 [.72, 1]**	.59 [.48, 1]	.56 [.46, 1]	.56 [.46, 1]	**.77 [.70, 1]**
**FSDS-DAO**	.72 [.63, 1]	.60 [.50, 1]	.58 [.47, 1]	.76 [.68, 1]	.77 [.68, 1]	.61 [.52, 1]	.56 [.46, 1]
**SIDI-F-SR**	**.72 [.64, 1]**	.54 [.44, 1]	.50 [.40, 1]	**.72 [.63, 1]**	**.77 [.68, 1]**	.56 [.46, 1]	.49 [.39, 1]

*Note.* Estimates in bold typeface = expected high associations due to overlap in the measured construct; brackets indicate one-sided 95% confidence intervals. IA = *Female Sexual Interest/Arousal Disorder*; I = *Hypoactive Sexual Desire Dysfunction*; A = *Female Sexual Arousal Dysfunction*; O = *Female Orgasmic Disorder* (DSM-5) or *Orgasmic Dysfunctions* (ICD-11); P = *Genito-Pelvic Pain/Penetration Disorder* (DSM-5) *or Sexual Pain-Penetration Disorder* (ICD-11).

#### Association of DISEX-F Diagnoses and FSDS-DAO Results

There was a significant positive association between the presence of an ICD-11 and DSM-5 diagnosis in the DISEX-F and the FSDS-DAO total score (ICD-11: AUC = .71 [.62, 1]; DSM-5: AUC = .68 [.57, 1]). However, the positive association between the FSDS-DAO total score and the presence of a DISEX-F diagnosis was not significantly higher for ICD-11 than for DSM-5 evaluation (*D* = 0.38, *p* = .351).

#### Association of DISEX-F Diagnoses and SIDI-F-SR Results

There was a significant negative association between the presence of a Hypoactive Sexual Desire Dysfunction (ICD-11) in the DISEX-F and the SIDI-F-SR total score (AUC = .72, CI [.63, 1]). Further exploration showed that this negative association was significantly higher than the association between the presence of Orgasmic Dysfunctions (ICD-11) and the SIDI-D-SR total score (*D* [193.93] = 1.96, *p* = .026) and higher than the association between the presence of Sexual Pain-Penetration Disorders (ICD-11) and the SIDI-F-SR total score (*D* [194.44] = 2.93, *p* = .002). However, the highest association was found for the Female Sexual Arousal Dysfunction (ICD-11) and the SIDI-F-SR total score (AUC = .77, CI [.68, 1]). For DSM-5 diagnoses, there was a significant negative association between the Female Sexual Interest/Arousal Disorder (DSM-5) and the SIDI-F-SR total score (AUC = .72, CI [.64, 1]). This association was significantly higher than the association between the SIDI-F-SR total score and (a) the presence of Female Orgasmic Disorder (DSM-5; *D* [190.68] = 2.26, *p* = .013) and (b) the presence of Genito-Pelvic Pain/Penetration Disorder (DSM-5) in the DISEX-F (*D* [192.63] = 2.81, *p* = .003).

### Patient Acceptance of the DISEX-F

Participant responses from the PAQ indicate a generally high level of acceptance of the DISEX-F. On the scale measuring global satisfaction with the interview, the mean score was 95%. Specifically, all participants experienced the relationship with the interviewer as pleasant, 95% of participants experienced the approach of the interview as helpful, 90% felt taken seriously with their problems, and 92% stated that they had been able to report everything that truly moved them (completely or almost completely agreed; displaying content evidence of validity). Only a small percentage of participants completely or almost completely agreed to feel interrogated (3%), confused (3%), or too exhausted by the interview (1%). Even though it was not the primary purpose and goal of the interview conduction, 20% even reported to understand their problems better after the interview (see Table 5). We explored whether there were meaningful differences in terms of acceptance ratings comparing women with (*n* = 85) and without ICD-11 and/or DSM-5 diagnoses (*n* = 15). Furthermore, we explored whether age as well as the duration of the initial interview were associated with acceptance ratings. None of these analyses revealed significant results.

**Table 5:** Mean, Standard Deviation, and Distribution of Answers of the Items of the Patient Acceptance Questionnaire (Suppiger et al., 2009; N = 100).

**Items^a^**		**Distribution of given answers**			
	*M (SD)*	0	1	2	3
**Positively formulated items**					
I experienced the approach of the interviewer as helpful.	2.59 (0.62)	1%	4%	30%	65%
I feel like the interviewer takes my problems seriously.	2.52 (0.88)	8%	2%	20%	70%
I found the relationship with the interviewer to be pleasant.	2.74 (0.44)	0%	0%	26%	74%
I think the interviewer asked detailed questions to better understand my situation.	2.55 (0.66)	2%	3%	33%	62%
I have the feeling that I understand myself and my problems better after the interview.	0.98 (0.86)	30%	50%	12%	8%
**Negatively formulated items**					
I feel more confused than before the interview.	0.1 (0.39)	93%	4%	3%	0%
I feel interrogated.	0.19 (0.51)	85%	12%	2%	1%
There were too many questions for me.	0.09 (0.29)	91%	9%	0%	0%
The interview was too exhausting.	0.13 (0.37)	88%	11%	1%	0%
I did not bring up everything that truly moved me today.	0.36 (0.75)	76%	16%	4%	4%

*Note*: Items were rated on a 4-point scale ranging from 0 to 3 (0 = *disagree*, 1 = *slightly agree*, 2 = *almost completely agree*, 3 = *completely agree*).

a Translated from the original German items printed in Behavior Therapy, 40/3, Andrea Suppiger, Tina In-Albon, Stephanie Hendriksen, Ernst Hermann, Jürgen Margraf, Silvia Schneider, Acceptance of Structured Diagnostic Interviews for Mental Disorders in Clinical Practice and Research Settings, 272–279, 2009, with permission from Elsevier.

## Discussion

The present study aimed to gather first data on the reliability and validity of the DISEX-F in a sample of symptom-reporting women. To this end, next to re-examining inter-rater reliability (see Schwesig et al., 2023a), test-retest reliability as well as convergent and discriminant evidence of validity of the DISEX-F were investigated for the first time. Furthermore, we collected first data on the acceptance of the DISEX-F among women experiencing sexual problems.

### Reliability And Source of Diagnostic Discordance

Inter-rater reliability of the DISEX-F was excellent for all sexual dysfunctions in both classification systems. All lower limits of the CIs were above the predetermined threshold. The Hypoactive Sexual Desire Dysfunction, the Female Sexual Arousal Dysfunction and the Orgasmic Dysfunctions of the ICD-11 showed perfect agreement (κ≥.99). Estimates were slightly lower for the Sexual Pain-Penetration Disorder (ICD-11, κ = .87) as well as for all DSM-5 disorders in the DISEX-F (κ≤ .94). Notably, lower estimates were found for exactly those diagnoses that require an additional judgment about the etiology of the sexual problem. As argued by Schwesig et al. (2023a), those diagnoses are probably more susceptible to diagnostic errors and diagnostic disagreements. This assumption is supported by subsequent analyses of mismatch which identified divergent differential diagnostic decisions as a major source of mismatch for those disorders. Divergent differential diagnostic decisions were especially frequent for the Sexual Pain-Penetration Disorder (ICD-11) and the Genito-Pelvic Pain/Penetration Disorder (DSM-5). Results are in line with findings from Schwesig et al. (2023a) who identified incorrect differential diagnostic decisions (compared to a predetermined diagnoses of a case vignette) as one of the major sources of mismatch when using the DISEX-F (it accounted for 43.8% of mismatch for DSM-5 diagnoses). In case of the Female Sexual Interest/Arousal Disorder (DSM-5), another source of mismatch was striking: Three out of four cases of mismatch appeared as diagnosticians wrongly assigned the dysfunction although only one or two of the required three symptoms were present. These slips might appear because of the unique requirement for the presence of three symptoms (rather than the usual one) for diagnostical assignment. Compared to the initial validation study by Schwesig et al. (2023a), all estimates of inter-rater reliability were found to be higher in the present study. This can clearly be traced back to differences in the research design: In the present study, patient-side information was held constant due to the use of audio records. In the initial validation study, estimates were calculated from two distinct interview conductions carrying the potential for information variations.

In the present study, estimates of test-retest reliability of the DISEX-F were lower than the estimates for inter-rater reliability. This was to be expected since the present test-retest reliability design introduces more sources of variance, for example, variations in the patient reports, different interviewing styles of the diagnosticians, and changes of the patient’s health status over time (for comparison, see Grove et al., 1981). However, computed estimates were still in a clinically acceptable range (ICD-11: κ = .66–.79, DSM-5: κ = .69–.75) with all lower CI limits equal or above .57. Furthermore, the magnitude of the estimates found is comparable to the magnitude of test-retest reliability estimates found in other validation studies of structured interviews (e.g., Hoyer et al., 2020; Osório et al., 2019; Schneider et al., 1992; Shabani et al., 2021; Suppiger et al., 2008; Williams et al., 1992). Interestingly, while the Genito-Pelvic Pain/Penetration Disorder (DSM-5) and the Sexual Pain-Penetration Disorder (ICD-11) exhibited the highest test-retest reliability, the Female Sexual Interest/Arousal Disorder (DSM-5) and the Hypoactive Sexual Desire Dysfunction (ICD-11) demonstrated the lowest test-rest reliability. Subsequent in-depth analyses of mismatches showed that divergent differential diagnostic decisions also reduced the diagnostic agreement concerning test-retest-reliability: 22.0% of mismatch of DSM-5 dysfunctions and 7.8% of mismatch of ICD-11 dysfunctions were attributable to this source of mismatch. Taken together, diverging differential diagnostic decisions were found as major source of mismatch for inter-rater reliability as well as test-retest reliability in the present study and as a major source of mismatch for sensitivity and specificity of the DISEX-F in the initial validation study (see Schwesig et al., 2023a). This clearly emphasizes that the requirement to judge the etiology of a sexual problem inevitably poses a higher risk of diagnostic disagreement. In addition, it also seems to place increased demands on the diagnostician. Therefore, these empirical findings demonstrate the need to diagnose those sexual dysfunctions within a broader diagnostic process. The process should include a multi-professional team, a medical consultation report to identify the influence of medical conditions and medication, as well as a full diagnostic clarification of the existence and influence of psychiatric disorders on the sexual problem. Such comprehensive diagnostics should enhance the diagnostic agreement on the concerned sexual dysfunctions.

Nevertheless, not divergent differential diagnostic decisions but information variance in participants reporting was identified as the major drive of diagnostic discordance. It accounted for 73.2% (DSM-5) and 92.2% (ICD-11) of mismatch. Information variance seems to generally limit the realistic expectancy values of test indices as it was also identified as a major source for discordant diagnostic outcome by validation studies of other structured interviews (e.g., Suppiger et al., 2008). The found variance in participants’ responses highlight the complexity of assessing symptoms related to sexual health and shows that perceiving and integrating sexual problems seems to be a process with at least some fluctuation. These findings are in line with observations about the “waxing and waning of symptoms” of mental disorders, describing the improvement and worsening of symptoms over time depending on stress and context (compare Katerndahl et al., 2005 and Wittchen et al., 2000). Depending on the point at which a patient is questioned, the perceived symptoms may or may not reach the diagnostical threshold necessary for a clinical diagnosis.

In the present study, the largest amount of information variance was found for sexual dysfunctions related to desire, arousal, and orgasm. This suggests that reported symptoms and distress related to desire, arousal, and orgasm may be less stable than experienced symptoms and distress related to sexual pain. Compared to the initial interview, there was a notable recurrent pattern of markedly increased symptom severity and distress reported at the second interview. This led to the phenomenon of more diagnoses being assigned at the second interview than at the first interview. One reason for this phenomenon may be that the questions of the first interview might have provoked a negative attentional bias. This in turn might have led to divergent (mostly enhanced) perceiving and reporting of symptom severity, frequency, and burden in the second interview. Another reason could be a familiarization with the interview situation resulting in less shame and more openness and honesty during the second interview. Clinical consequences of both hypotheses are (a) to motivate patients to track symptoms beforehand (self-observation), for example, by keeping a symptom diary to obtain realistic information on symptom severity in a subsequent diagnostic interview, and (b) to pay even more attention to creating an atmosphere of trust before and during the interview conduction.

### Convergent and Discriminant Evidence of Validity

Present results provide convergent evidence of validity of the DISEX-F and the underlying ICD-11 and DSM-5 diagnoses. As expected, the presence of ICD-11 and DSM-5 diagnoses in the DISEX-F was significantly associated with the results of the FSFI and FSDS-DAO. However, there was one exception: Contrary to expectations, DSM-5 diagnoses of the DISEX-F were not significantly associated with the FSFI results. This finding, however, may rather not be attributable to poor performance of the DISEX-F interview for DSM-5 diagnoses, but rather to the obvious low fit between the DSM-5 criteria and the questions of the FSFI. For example, while the DSM-5 requires 6 months of symptom duration, the FSFI only asks about the past 4 weeks. While the etiology of symptoms plays a decisive role for the diagnostical assignment in the DSM-5, none of the self-report questionnaires ask about the etiology. This potentially leads to situations, where people score low on the FSFI due to low sexual functioning, but still do not receive a diagnosis according to DSM-5 criteria due to, for example, organic origin of the sexual problem. The association between DISEX-F diagnoses and the FSFI and FSDS-DAO results was descriptively and, in case of FSFI, also significantly higher for ICD-11 than for DSM-5 evaluation. This, again, reflects the fact that the additional exclusion criteria of the DSM-5 lead to a weaker association with the self-report results.

Further exploratory analyses revealed first support for convergent and discriminant evidence of validity of the DISEX-F on a disorder level: Especially strong associations were found for DISEX-F diagnoses and FSFI subscales describing similar constructs, that is, the same sexual problem. Descriptively weaker associations were found for DISEX-F diagnoses and FSFI subscales describing different sexual problems. A subsequent reanalysis of the associations in a subsample of only sexually active women resulted in an elevation of all estimates in case of construct-related diagnoses and FSFI subscales. This again further indicates that the 4-week period inquired about in the FSFI reduces the to-be-expected association between the DISEX-F diagnoses and the FSFI results. Furthermore, as expected, especially the presence of the Hypoactive Sexual Desire Dysfunction (ICD-11) but also the presence of the Female Sexual Arousal Dysfunction (ICD-11) and the Female Sexual Interest/Arousal Disorder (DSM-5) in the DISEX-F were strongly associated with the SIDI-F-SR total score. As the SIDI-F-SR is a measure of the severity of Hypoactive Sexual Desire Dysfunction but also includes items assessing sexual arousal, results add to the convergent evidence of validity of DISEX-F diagnoses. As subsequent analyses showed, all other ICD-11 and DSM-5 dysfunctions in the DISEX-F were less strongly associated with the SIDI-F-SR, which adds to the discriminant evidence of validity of the DISEX-F diagnoses.

### Interview Acceptance

Particularly of note for the practice of the diagnostic process is that the results indicate a high level of acceptance of the DISEX-F among participants. Only few participants reported a negative impact of the interview situations such as feeling interrogated. In contrast, almost all participants reported that they experienced the relationship with the interviewer as pleasant, that they felt taken seriously, and were able to express what was on their minds. Although it is not its original goal, the DISEX-F interview process even seems to promote a kind of self-understanding as indicated by the participants. These findings clearly contradict common concerns about the potentially disruptive or intrusive nature of structured interviews, for example, interference with the therapeutical relationship. Instead, they underpin previous research on the actual experience of patients showing that structured interviews are well accepted and that the positive impact and the actual experience of patients are often better/higher than expected by therapists (Hoyer et al., 2006; Marshall et al., 2001; Neuschwander et al., 2017; Suppiger et al., 2009). With an average time of 38 min, conducting the DISEX-F is time-consuming. However, considering that the DISEX-F queries all criteria of the ICD-11 and DSM-5 and collects therapy-relevant information concerning the development and maintenance of the sexual problem, it is a time-efficient diagnostical tool.

### Limitations and Future Directions

Our research has several limitations: First, the study sample was community-based, that is, women with self-reported sexual problems were recruited from the general population. Thus, the DISEX-F was not validated in a clinical sample. However, such a sample (exclusively including people with clinically significant levels of sexual problems) would likely lead to biased estimates of the psychometric properties of the DISEX-F (e.g., an overestimation of reliability). In addition, it does not represent the sample the DISEX-F was mainly constructed for. We argue that our sample has close similarities with treatment-seeking samples presenting in outpatient clinics: Both samples present with self-reported sexual problems. They share the motivation to talk about their sexuality due to hope for a comprehensive diagnostical assessment, clinical feedback, information, and orientation. Furthermore, like clinical samples, the study sample represents a particularly vulnerable part of the population as indicated by high comorbidity and prevalence rates for mental disorders in the present sample (Jacobi et al., 2015). Nevertheless, the study sample might differ from treatment-seeking samples as the commitment for possible subsequent treatment remains unclear. Women might have participated for information purpose only and might not seek or consider treatment for their sexual problems. On the other hand, symptom-reporting women who seek treatment might have not participated in our study as we offered clinical feedback and information but not treatment. In addition, our study participants may have been particularly interested in the new interview and its results, which could have led to an upward bias in the acceptance ratings. The acceptance of the instrument might be lower when applied in routine care. Thus, to verify the psychometric properties of the DISEX-F when used in routine care, we suggest replicating the study in a treatment-seeking sample.

Second, diagnosticians were not explicit experts in the field of sexual dysfunctions and had overall limited clinical experience in diagnosing and treating sexual dysfunctions. As a consequence, results should provide a good indication of the psychometric properties of the DISEX-F for its main target group, that is, trained but not necessarily specialized clinical staff (for comparison, see Schwesig et al., 2023a). However, the present results most likely represent lower bound estimates of the reliability and validity of the DISEX-F than what would be expected when administered by experts in sexual dysfunctions. Furthermore, all diagnosticians were specially trained in conducting the DISEX-F. Untrained diagnosticians may obtain poorer validity and reliability estimates. Furthermore, three of four diagnosticians had also participated in the initial study and were experienced in using the DISEX-F. It could be argued that this explains the low rate of interview errors and that the found estimates are not realistic and too high for trained but unexperienced DISEX-F users. Nevertheless, a low rate of interview errors was also found for diagnosticians who were trained but unexperienced in using the DISEX-F in the initial validation study by Schwesig et al. (2023a). This indicates that the DISEX-F is easy to learn and hardly prone to interview error. As acceptance ratings might also be affected by the gender of the diagnosticians, it is important to note that we only included female diagnosticians in this study. However, since the interviews were conducted by four distinct female diagnosticians, it can be ruled out that the favorable reactions to the structured interview are solely attributable to the style and personality of one individual diagnostician. Despite efforts to ensure anonymity, there is a possibility that participants may have been influenced by social desirability when responding to the questions regarding the acceptance of the DISEX-F. Nevertheless, this concerns all comparable investigations (Neuschwander et al., 2017; Schwesig et al., 2023a; Suppiger et al., 2009).

Third, the time period between the first and the second interview varied between participants. While we aimed for a time period of 7 to 14 days, 18% of participants fell outside this time period, and few had even more than 20 days in between study sessions. Long time intervals might have further promoted the information variance given by the participants and reduced test-rest reliability. However, shorter intervals (below 7 days) could have increased kappa estimates.

Fourth, a common issue with the use of audio records for examining inter-rater reliability is that interviewers may unintentionally signal observers how they are rating the participant. For instance, the interviewer’s choice of wording for specific questions and his or her decisions such as skipping questions or sections when a key question is answered in a particular manner can inadvertently reveal their thoughts (see Grove et al., 1981). Furthermore, the use of audio records artificially reduces sources of diagnostical disagreement such as information variance in patient reporting or different interview styles. Altogether, this might have potentially resulted in artificially increased estimates of diagnostical agreement.

Together with the results from the initial validation study of the DISEX-F (Schwesig et al., 2023a), it can be concluded that there is stable evidence on the reliability and validity of the DISEX-F for both ICD-11 and DSM-5 diagnoses. Its validity and reliability were proven under ideal, standardized conditions using a vignette-based validation approach (see Schwesig et al., 2023a), as well as among women with sexual problems from the general population as indicated by the present study. As the DISEX-F has not been validated in routine clinical settings yet, we suggest its further validation in inpatient and outpatient clinical settings.

## Conclusion

The present study marked the second step in the validation process of the DISEX-F, that is, the examination of its psychometric properties in a sample of women experiencing self-reported sexual problems. Overall, the present results confirm the validity and reliability of the DISEX-F for both ICD-11 and DSM-5 diagnoses and its high acceptance among participants. Since the sample was community-based and the diagnosticians were experienced research interviewers, the DISEX-F should be further validated in inpatient and outpatient clinics to validate its psychometric properties in routine care. Overall, the present findings encourage the use of the DISEX-F as a diagnostical tool in clinical as well as research settings. It underpins its potential to enhance reliability and validity of clinical diagnoses, thereby counteracting non-replicability of research samples and incomparability of research findings.

## Supplemental Material

sj-docx-1-asm-10.1177_10731911241253659 – Supplemental material for Psychometric Properties of the Diagnostic Interview for Sexual Dysfunctions in Women in a Symptom-Reporting Sample Supplemental material, sj-docx-1-asm-10.1177_10731911241253659 for Psychometric Properties of the Diagnostic Interview for Sexual Dysfunctions in Women in a Symptom-Reporting Sample by Rebekka Schwesig, Maike Borchardt, Julia Velten and Jürgen Hoyer in Assessment
