# Supplementary material for: Psychometric Properties of the Diagnostic Interview for Sexual Dysfunctions in Women in a Symptom-Reporting Sample
Source: Assessment. 2024 Jun 3;32(4):590–607. doi: 10.1177/10731911241253659 (PMC12009447; doi:10.1177/10731911241253659)
Supplement: sj-docx-1-asm-10.1177_10731911241253659 – Supplemental material for Psychometric Properties of the Diagnostic Interview for Sexual Dysfunctions in Women in a Symptom-Reporting Sample [file sj-docx-1-asm-10.1177_10731911241253659.docx]

**Supplements**

**Table S1**

*Sample Characteristics: Demographic Data and Sexual Activity in the Past 12 Months*

| **Age** | |  | **Occupation** |  |
| --- | --- | --- | --- | --- |
| ﻿Mean | | 36.36 | Full-time occupation | 40% |
| Standard Deviation | | 12.21 | Part-time occupation | 12% |
| **Sexually attracted by** | |  | Student | 33% |
| Men (heterosexual) | | 68% | Other (e.g., parental leave, retired) | 15% |
| Women (homosexual) | | 2% | **Frequency of masturbation** |  |
| Men and women (bisexual) | | 17% | Not at all | 14% |
| Humans independent from gender | | 13% | Less than once per month | 27% |
| **Relationship status** | |  | 1-2 per month | 40% |
| ﻿ Monogamous relationship | | 75% | 1-2 per week | 16% |
| Single | | 15% | 3-4 times a week | 1% |
| ﻿Other (e.g., consensual nonmonogamy) | | 10% | More than 4 times a week | 2% |
| **Menopause status** | |  | **Frequency of partnered sex** |  |
| ﻿Premenopausal | | 79% | Not at all | 13% |
| Perimenopausal | | 11% | Less than once per month | 25% |
| Postmenopausal | | 10% | 1-2 per month | 42% |
| **Education** **(highest degree)** |  | | 1-2 per week | 17% |
| No degree | | 0% | 3-4 times a week | 3% |
| Lower school | | 1% | **Children (Yes)** | 43% |
| Middle school | | 11% |  |  |
| A-Levels | | 33% |  |  |
| Completed apprenticeship/masters training | | 8% |  |  |
| University degree | | 44% |  |  |
| PhD | | 3% |  |  |

**Table S2**

*Number of Diagnoses Assigned After the First and Second Interview*

|  | **Number of diagnoses** | |
| --- | --- | --- |
|  | First interview | Second interview |
| **DSM-5** |  |  |
| Female Sexual Interest/Arousal Disorder | 39 | 42 |
| Female Orgasmic Disorder | 40 | 48 |
| Genito-Pelvic Pain/Penetration Disorder | 39 | 37 |
| **ICD-11** |  |  |
| Hypoactive Sexual Desire Dysfunction | 29 | 43 |
| Female Sexual Arousal Dysfunction | 27 | 32 |
| Orgasmic Dysfunctions | 43 | 51 |
| Sexual Pain-Penetration Disorder | 37 | 37 |

**Table S3**

*Convergent and Discriminant Evidence of Validity: AUC Values of the ROC Curves for the DISEX-F Diagnoses and the Results of the Self-Report Measures of Sexual Functioning and Sexual Distress Only Including Women with no Zero Categories in the FSFI (n* *=70).*

|  | **DSM-5** | | | **ICD-11** | | | |
| --- | --- | --- | --- | --- | --- | --- | --- |
|  | **IA^1^** | **O^4^** | **P^5^** | **I^2^** | **A^3^** | **O^4^** | **P^5^** |
| **FSFI**-Desire | **.77 [.69, 1]** | .55 [.43, 1] | .70 [.60, 1] | **.70 [.58, 1]** | .60 [.48, 1] | .56 [.45, 1] | .66 [.56, 1] |
| **FSFI**-Arousal | **.62 [.50, 1]** | .65 [.55, 1] | .50 [.37, 1] | .61 [.49, 1] | **.74 [.64, 1]** | .65 [.55, 1] | .50 [.37, 1] |
| **FSFI**-Lubrication | **.63 [.51, 1]** | .58 [.47, 1] | .46 [.35, 1] | .56 [.43, 1] | **.77 [.66, 1]** | .62 [.50, 1] | .47 [.35, 1] |
| **FSFI**-Orgasm | .58 [.46, 1] | **.83 [.75, 1]** | .52 [.39, 1] | .63 [.51, 1] | .43 [.31, 1] | **.86 [.78, 1]** | .50 [.36, 1] |
| **FSFI**-Pain | .60 [.49, 1] | .59 [.48, 1] | **.89 [.83, 1]** | .55 [.41, 1] | .53 [.40, 1] | .54 [.43, 1] | **.90 [.84, 1]** |

*Note.* Estimates in bold typeface = expected high associations due to overlap in the measured construct; brackets indicate one-sided 95% confidence intervals. ^1^IA= *Female Sexual Interest/Arousal Disorder;* ^2^I = *Hypoactive Sexual Desire Dysfunction;* ^3^A= *Female Sexual Arousal Dysfunction;* ^4^O= *Female Orgasmic Disorder* (DSM-5) or *Orgasmic Dysfunctions* (ICD-11)*,* ^5^P= *Genito-Pelvic Pain/Penetration Disorder* (DSM-5) *or Sexual Pain-Penetration Disorder* (ICD-11).
